# Capturing more than a snapshot: Methodological considerations and the development of an analytical framework for longitudinal interpretative phenomenological analysis (LIPA)

**DOI:** 10.1111/bjhp.70114

**Published:** 2026-10-04

**Authors:** Joseph Keenan

**Affiliations:** ^1^ School of Psychology Manchester Metropolitan University Manchester UK

**Keywords:** experiential trajectories, longitudinal interpretative phenomenological analysis, longitudinal qualitative research, phenomenology

## Abstract

The use of longitudinal approaches when conducting qualitative research is becoming increasingly commonplace. From a phenomenological perspective, longitudinal research aims to generate knowledge by placing an enhanced interpretative focus on temporality when exploring how individuals make sense of their experiences to generate meaning. This paper provides an overview of some practical and methodological considerations that researchers must consider when using longitudinal interpretative phenomenological analysis. The considerations covered in this paper include (i) acknowledging the flexibility and complexity of using LIPA, (ii) embedding creative methodologies, (iii) minimizing attrition, (iv) ensuring sustained ethical research practices and (v) tracking individual and group trajectories when analysing an experientially rich and large data corpus. This paper also provides a flexible and adaptive framework of analytical stages that researchers may adopt when seeking to interpret longitudinal experiential data. The paper concludes with a balanced overview of the strengths and limitations of adopting a longitudinal approach within phenomenological research.

**Statement of Contribution**

**What is already known?**

- Longitudinal qualitative research is becoming increasingly used by health researchers to explore continuity and change in a variety of contexts.
- Longitudinal interpretative phenomenological analysis (LIPA) is a useful analytical tool to explore temporal experiential sense‐making in health contexts
- When using LIPA, researchers must make a series of complex decisions relating to both design and methodological application.

**What does this study add?**

- This article suggests a flexible analytical approach that researchers can adopt when using LIPA to interpret longitudinal experiential data and identify experiential trajectories.
- This article provides readers with a case exemplar of how LIPA can be explored to investigate trajectories of sense‐making in the context of long‐term recovery from substance use.
- This article offers practical guidance to readers considering using LIPA concerning embedding creative approaches, strategies for minimizing attrition and ensuring ethical practice in prolonged researcher projects.

## INTRODUCTION

Traditional methods of qualitative analysis often focus on interpreting meaning at a single point in time (Smith, 2024). However, this sometimes fails to capture the complex, dynamic and iterative way people navigate the communities to which they belong and their lived experiences. Longitudinal interpretative phenomenology analysis (LIPA) is an innovative approach that allows researchers to explore how their participants navigate and create meaning from their experiences over a temporal period. Like traditional phenomenological inquiry, LIPA is underpinned by central tenets, including double hermeneutics, intersubjectivity and retaining focus on an idiographic level of analysis. LIPA differentiates itself from more traditional forms of IPA through its enhanced focus on temporal trajectories, allowing researchers to explore the stability, dynamism and evolution of a person's meaning‐making over time. It is argued that people dynamically reflect on their past, present and anticipated futures and use these reflections to generate and shape meaning from their lived experiences (Smith, 2024). This representation of dynamism, in the way individuals make sense of the world, provides an opportunity within health research to explore more temporally how individuals navigate their health, well‐being and identities throughout the life course. LIPA differs from alternative forms of longitudinal qualitative analysis, such as Biographic Narrative Interpretation Method (BNIM; Wengraf, 2008), as it does not seek to reconstruct the personal and social history of an individual. Moreover, LIPA aims to understand the essence and meaning of a specific phenomenon for individuals. From a more longitudinal perspective, BNIM places temporal focus on the historical unfolding of an individual's life, whereas LIPA places a greater focus on change and continuity in an individual's sense‐making of a particular experience over time.

Early examples of longitudinal phenomenological research conducted in health contexts include Smith (1999), who explored how first‐time mothers navigated pregnancy and their transition into motherhood. Through a series of detailed case studies, Smith (1999) identified the dynamic way in which mothers transitioned from navigating adjustment and questioning (in early pregnancy) to a sense of ambivalence, impatience and anxiety (in late pregnancy) and finally to the readjustment to the presence of their child and the impact of motherhood on life priorities (post‐partum). The use of LIPA in this study represents its potential usefulness in health‐related research through the provision of a temporal understanding of the dynamic processes that people may encounter during their lives.

Like the transition to motherhood, adapting to living with a long‐term illness represents a dynamic health‐related milestone in a person's life. Researchers (Chan et al., 2017; Snelgrove et al., 2013; Tedlie Moskowitz & Wrubel, 2005) have used variations of LIPA to explore how people navigate and make sense of living with a variety of long‐term conditions, such as chronic lower back pain, psoriasis and HIV. Each of these studies emphasizes psychological adaptations and transitions that individuals experience, such as learning to cope with symptoms, stigmatizing responses from communities and making adjustments that are needed to fulfill occupational, familial and social roles. More recently, researchers have utilized LIPA to explore trajectories of meaning‐making in other clinical populations such as exploring how mood changes over time for people living with bipolar disorder (Farr et al., 2025). Farr et al. (2025) identified how changes in meaning‐making of participants' experiences of agency and connectivity contribute to fluctuating perspectives of future direction. The authors helpfully distinguish these dynamic constructions of meaning and how they manifest across periods of depression, mania and emotional stability. Similarly, LIPA has been adopted to explore concepts such as post‐traumatic growth in people admitted to intensive care units and subsequently discharged to receive care in community settings (Li et al., 2025). Li et al. (2025) argue that findings of LIPA studies can be used to help clinicians consider how aftercare services are implemented to support patients who are dynamically navigating a period of trauma response, growth, collapse and long‐term recovery.

Despite LIPA's comparable infancy to other forms of longitudinal qualitative approaches, contemporary researchers have started to identify some important methodological issues that researchers must consider when conducting a LIPA (Farr & Nizza, 2019; Snelgrove et al., 2013; Wanat et al., 2025). Snelgrove et al. (2013) provided some helpful early considerations by identifying some common pitfalls of using LIPA, including the difficulties of maintaining an inductive approach and challenging the usefulness of bracketing. Farr and Nizza (2019) conducted a review of studies that had utilized LIPA to identify common methodological features of high‐quality studies. They synthesized key methodological decisions made by the authors of included studies concerning *timing*, *approaches to data collection* and *how findings were presented*. The timing of synchronous data collection is key. The authors distinguish between prospective and retrospective forms of data collection and the sometimes challenging task of identifying dynamism when data is collected within short interval time points. In relation to data collection, Farr and Nizza (2019) identified that most LIPA studies used semi‐structured interviews as a data collection method and discussed the advantages and disadvantages of using similar topic guides/interview schedules at each point of data collection or creating specific versions for each point of data collection. In sum, they argue that due to the generally large amount of data collected in LIPA studies, researchers should prioritize choosing an approach that enables them to fulfill their research objectives. Finally, Farr and Nizza (2019) found that in a substantial minority of LIPA papers, researchers relied on either the use of a generic citation linked to more traditional forms of IPA or communicated their findings in a manner that lacked idiography or did not clearly identify the longitudinal elements of their findings. Farr and Nizza helpfully identified two effective ways that researchers used to communicate findings, distinguishing between *themes spanning time* and *themes tied to time points*. Wanat et al. (2025) add that IPA's roots in phenomenology, hermeneutics and idiography make it particularly suited to exploring temporal dimensions of experience. However, they provide a cautionary note that applying a longitudinal lens to phenomenological research goes beyond collecting data more than once, and that researchers seeking to conduct a LIPA must make a series of complex methodological decisions throughout the entirety of the research cycle.

## RATIONALE AND PURPOSE

Despite the growing use of longitudinal phenomenological approaches in health research, a paucity of practical guidance remains on how to overcome common methodological pitfalls and to conduct the analysis. As such, this paper will serve as a practical guide that researchers can use when conducting longitudinal phenomenological research. When implementing multiple time points to a phenomenological inquiry there are several practical methodological considerations and questions that the researcher must attend to, including (i) acknowledging the flexibility and complexity of using LIPA to identify the temporal scope of a research study, (ii) how to embrace and embed creative methodologies within longitudinal phenomenological research, (iii) how to minimize attrition in longitudinal phenomenological research, (iv) how to construct iterative and tailored interview schedules and topic guides, (v) how to ensure your research retains ethical integrity throughout the duration of the project and (vi) how to track individual and group trajectories when analysing an experientially rich and large data corpus.

As such, this paper aims to (i) utilize worked examples to guide both novice and established longitudinal qualitative researchers on how they may respond to the above methodological questions, (ii) highlight how LIPA can be used in a variety of health‐related contexts and (iii) provide a flexible step‐by‐step analytical approach that can be used to guide researchers who are planning to conduct a LIPA.

## PRACTICAL CONSIDERATIONS FOR RESEARCHERS

### Flexibility and complexity

Conducting a LIPA is complex, as both participants and researchers can engage in both prospective and retrospective forms of sense‐making at either singular or multiple time points. As such, researchers must consider the temporal scope and focus of their study when considering decisions such as the immediacy of points of data collection, tracking individual trajectories, the selection of appropriate data to identify change or continuity, and deciding whether change should be explored at an individual level, group level or both (Farr & Nizza, 2019). Such flexibility allows researchers to design studies that can explore hermeneutic sense‐making in the period leading up to a specific phenomenon and subsequently following it (e.g., expectant parents’ transition into parenthood). Alternatively, researchers may wish to track individual or group trajectories of sense‐making over a prolonged period, focusing on an enduring phenomenon such as long‐term recovery from addiction or coping and adaptation to living with a long‐term health condition.

Based on the temporal focus of individual studies, researchers may choose to schedule interviews before and after a specific event occurs to increase the levels of immediacy or take a more retrospective approach that includes longer intervals between points of synchronous data collection. To demonstrate the flexibility of how LIPA may be used, I will draw upon examples from my own research (Cornwall, 2025; Keenan et al., 2026; Rahman et al. 2020). In both studies, the focus of the study was to explore both group and individual trajectories of hermeneutic sense‐making across the duration of the study.

In one application of LIPA, we (Rahman et al., 2020) explored how palliative care patients navigated their experiences of accessing psychosocial art‐based therapy through a videoconferencing platform. Each participant in the study was introduced to the videoconferencing software simultaneously and subsequently interviewed at three monthly intervals during the project. At a group level during the first set of interviews, we observed that all participants experienced anxiety as they learned how to use the technology. However, during later interviews (second and third interviews), participants developed a sense of mastery in using the equipment and focused more on how it enabled them to build and sustain strong therapeutic relationships with healthcare professionals. In this example, the advantage of using LIPA with a group of people experiencing something new for the first time allowed us to explore how participants experientially navigated the introduction of a digital therapeutic intervention during relatively short intervals. In addition, we were also able to track idiographically how each participant made sense (both prospectively and retrospectively) of accessing therapeutic support through digital methods and how this created meaning in relation to their individual psychosocial needs at the end of life over the duration of the study.

In contrast to the above study, we (Cornwall, 2025; Keenan et al., 2026) used a more temporally fluid LIPA approach, with longer intervals between data collection points. This study aimed to explore how participants made sense of their experience of long‐term recovery from substance misuse. The project involved 20 participants, all at various stages of long‐term recovery (ranging from 3 to 29 years), who were interviewed twice, with an interval of 1 year between interviews. Adopting LIPA in this way allowed us to place greater focus on the trajectories of each participant and explore how they created meaning experientially. Through this application, we were able to identify conceptual processes that people idiographically navigated during their recovery. At a group level, participants described a process of transitioning from a temporal pattern of introspection, progressing through a process of maturation, before reaching a level of comfort and authenticity in relation to their self‐concept and the relationships they shared with others. Moreover, the process was grounded within their social context and resources (intrinsic and extrinsic) available to each individual.

As the above examples suggest, one of the key benefits of using LIPA in health research is its flexibility of implementation and adaptability in meeting research objectives. It allows researchers to explore the dynamic (or static) way in which groups or people navigate their experiential trajectories in health contexts. However, with such flexibility, researchers must consider the scope of their research question(s) and ascertain whether a LIPA or a more traditional approach to phenomenological inquiry is most appropriate. For instance, the researcher may wish to explore how a person makes sense of their experience at a specific point of an illness trajectory (i.e., diagnosis) or take a more holistic approach to understanding an illness trajectory (i.e., coping and adaptation to living with a long‐term condition). In the first instance, it would not make much sense to conduct a LIPA, as the scope of the project is solely focused on one fixed temporal point. However, in the latter example, there are many factors which may influence how an individual makes sense of their illness and adapts over time. For example, an individual may make sense of their illness experience through an embodied sense of fear during the earlier stages of an illness (e.g., during the onset of physical symptoms), but upon adjustment, may interpret their illness experience as one which is underpinned by supportive relationships with health care professionals or hope for recovery (Martino et al., 2022).

Researchers should also consider the length of intervals between points of synchronous data collection when designing their LIPA study. For some LIPA studies, having relatively short (e.g., weekly or monthly) intervals between data collection points may enhance the richness of recall from participants (Farr & Nizza, 2019). However, researchers and participants may also struggle to identify and communicate change in their sense‐making (Farr & Nizza, 2019). In contrast, longer durations between data collection time points may arguably make it easier to identify changes in sense‐making for groups and participants over time. However, using the latter approach may also lead to a dilution of the richness of the data corpus, through the increased cognitive load involved for participants having to recall information across longer durations. I argue this decision should be informed by the wider research context in which LIPA is being used. For example, if a LIPA is being used as part of a process evaluation for a behaviour change or therapeutic intervention, it may be beneficial to position data collection intervals at relatively short intervals such as pre‐intervention, mid‐intervention and post‐intervention. This may allow the researcher to identify patterns of sense‐making more easily at both a group and an individual level at more specified time points. In contrast, longer intervals (e.g., a year) between data collection points may be beneficial when the temporal scope of a study focuses on a more long‐lasting or enduring phenomenon. A longer timeframe allows the researcher to interpret dynamic (and static) patterns more easily in the way individuals make sense of their experiences.

### Embracing and embedding creative methodological approaches when using LIPA

Creative methods provide an excellent way of elucidating rich and detailed responses from participants and are becoming increasingly popular within qualitative research. It provides an opportunity for individuals to express their views, perceptions and sense‐making in meaningful and visual ways (Kara, 2015; Rainford, 2020). It has been demonstrated that the use of elucidation techniques such as poetry (Szto et al., 2005), arts‐based (Broadbent et al., 2004, 2009) and photovoice (Catalani & Minkler, 2010) can be a successful strategy for participants to communicate their meaning‐making to researchers.

Adopting creative methods within longitudinal phenomenological research can provide an opportunity for participants to creatively navigate their sense‐making over time. For example, Bullo and Hearn (2024) embedded creative art‐based methods when investigating how women make sense of pain when living with endometriosis. Participants were asked to create models representing how they wanted to visualize their pain. In earlier sessions, pain was visually represented as complex and multifaceted artistic structures. However, as time progressed, the structures became increasingly smaller, representing how participants' temporal and embodied sense‐making changed as they navigated and compartmentalized their experiences of pain while living with endometriosis.

Multimodal approaches to data collection such as integrating arts‐based data alongside more traditional verbal data enable researchers to examine change and continuity across multiple dimensions of participant experience. While interview data capture participants' explicit reflections and narratives, arts‐based outputs may reveal emotional, embodied or tacit aspects of experience that are difficult to articulate verbally. From a longitudinal perspective, generating these forms of data across successive time points allows researchers to identify both stable themes and evolving perspectives.

Data integration occurs at both the analytic and interpretive stages. Arts‐based and verbal data should initially be analysed independently to identify visual, linguistic, symbolic and narrative components. During the interpretative stage of analysis, researchers can also pay close attention when attempting to identify whether meaning‐making occurs first in a specific approach (e.g., interpreted in a picture drawn by a participant before verbalizing the same concept in an interview), tracking levels of consistency between verbal accounts and artistic representations of meaning (e.g., participants discussing a clear understanding of the phenomenon being discussed but producing conflicting and complex representations of the topic). As such, multi‐modal sources of data can arguably provide the researcher with a richer understanding of developmental trajectories than either method alone.

### Minimizing attrition in longitudinal phenomenological research

Establishing trust and rapport with participants is important when undertaking any form of qualitative research, as it helps participants to speak openly during interviews and focus groups and provide rich data (Gagner, 2014; Gill et al., 2008; McGrath et al., 2019). Maintaining a strong level of trust and rapport with participants is even more important when conducting longitudinal forms of qualitative research due to the prolonged engagement between participants and researchers (Derrett & Colhoun, 2011; Grinyer & Thomas, 2012). Failure to establish trusting relationships with participants in an initial interview will increase the chance of attrition at future data collection points.

Several other motivating factors may lead to attrition in longitudinal studies, including time constraints, competing life demands, lack of flexibility when scheduling data collection or simply the loss of motivation to participate further in the research study (Parra‐Medina et al., 2004; Tansey et al., 2007). To minimize or avoid attrition in longitudinal research, there are several strategies that the researcher may wish to implement. Firstly, researchers can demonstrate that they have a strong understanding of the phenomena under investigation and demonstrate compassion and respect to their participants (Patel et al., 2003). Secondly, as suggested above, embedding creative activities that participants can engage with during asynchronous periods of a study, such as journaling, Photovoice or drawing, may help to minimize attrition. Finally, researchers may wish to schedule follow‐up calls with participants at an agreed interval to update participants on the progress of the research and to flexibly arrange the next interview (Ziek et al., 1996).

### Constructing iterative and tailored interview schedules and topic guides

When adopting a longitudinal phenomenological approach, researchers can be strategic in the way specific topics and questions are raised with participants. The initial phases of data collection should aim to provide the researcher with a rich understanding of the socio‐demographic context of the participant. The information provided by participants will allow the researcher to ensure that they can maximize the extent to which they can iteratively explore the phenomena under investigation at subsequent data collection intervals. From a practical perspective, some examples of specific questions which may be posed include: ‘How would you describe your current experiences of [topic]?’ If adopting a multi‐modal approach to data collection through the integration of an arts‐based approach, researchers may also prompt participants to engage with this activity by posing a question such as: ‘Can you create an image that represents where you see yourself at this point in time?’

For subsequent interviews, I argue that topic guides and prompts should be adapted and tailored to ensure that participants can discuss and reflect on any temporal changes in the way they make sense of their experiences during the interim period between interviews (Bolger & Laurenceau, 2013; Pereira et al., 2021). From a practical perspective, during subsequent points of data collection, researchers may wish to ask a question such as: ‘Looking back on our previous discussion, what has changed since then?’ If participants indicate that there has been change in the way they create meaning from their experience, the researcher may pose a follow‐up question such as: ‘What do you think has shaped the change in the way you feel about [topic]?’ If using a multi‐modal approach integrating arts‐based data collection techniques, researchers may utilize the visual representations and pose a question such as: ‘How does your new artistic representation compare with the one you created previously?’

In the final synchronous data collection period, researchers may wish to focus more holistically on participants' experiences and reflections across the entirety of their involvement in the study. From a practical perspective, questions at this stage may include: (i) ‘Reflecting across the whole study period, what changes stand out most?’ (ii) ‘What aspects of your experience have remained stable?’ and (iii) ‘If you could revisit your first interview, what would you tell your earlier self?’ If the researcher has incorporated an arts‐based approach, they may also utilize the creative outputs as an elucidation tool and ask participants a question like: ‘How do you interpret the similarities and differences among the creative outputs that you have produced at each stage?’

Figure 1 (below) provides a more detailed overview of how researchers can iteratively construct topic guides in the context of a study with three intervals of data collection.

**FIGURE 1:**
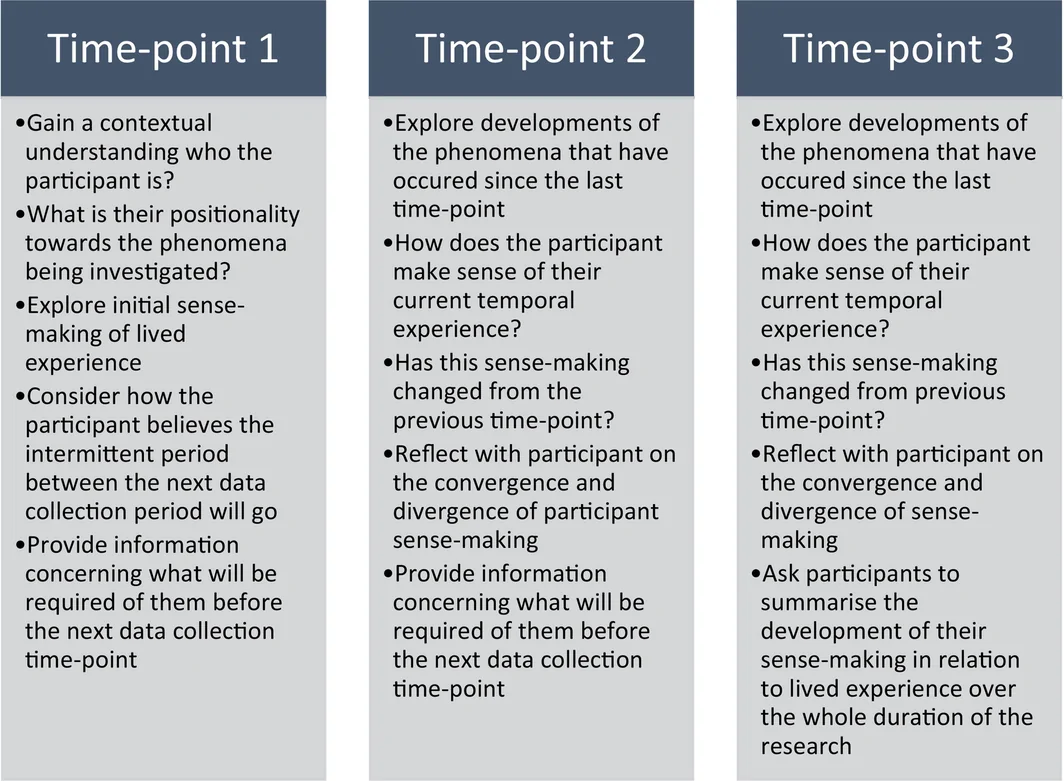
Key activities at each point of data collection – Example of a three‐time‐point design.

### Ensuring your research retains ethical integrity throughout the duration of the project

Like all research involving human participants, there are many important ethical issues that researchers must consider when conducting longitudinal phenomenological research. Practically, longitudinal phenomenological studies require participants to engage in a research process over a prolonged period and commit a greater amount of their time compared to a study that only has one point of data collection. To overcome this, researchers should clearly communicate the longitudinal aims of their research to participants and, where possible, offer remuneration for their time. Additionally, Calman et al. (2013) suggest that when using a qualitative design, the process of gaining consent should be iterative and completed at each data collection time point. Furthermore, participants should be able to withdraw from the process at any point during the project without consequence.

More conceptually, when conducting research into a sensitive topic (e.g., bereavement, coping with a long‐term illness), there is an increased chance that participants will continue to reflect on negative aspects of their experience during the prolonged research process. As such, researchers should ensure that their participants are adequately signposted to relevant sources of emotional support (e.g., relevant charities, healthcare professionals) that they can contact throughout the duration of the project and following the conclusion of their direct involvement in the research.

### Tracking individual and group trajectories when analysing an experientially rich and large data corpus

A core challenge facing researchers conducting a LIPA is keeping track of individual and group sense‐making across temporal trajectories. The added complexity of a longitudinal design increases the analytical load on researchers, as they try to maintain an idiographic approach across multiple accounts from participants over time. This increased analytical load can make it easier for researchers to lose sight of an individual's unique trajectory as temporal layers of the longitudinal analysis accumulate, due to the large volume and dense datasets that are produced in a LIPA study (Tuthill et al., 2020). Consequentially, it is easy for an analyst to become overwhelmed and inadvertently focus on salient or the most recent data point or focus solely on the interpreted group patterns at the expense of idiographically coherent, detailed longitudinal case histories. Tracking trajectories requires identifying change, continuity, or rupture in experience. This is challenging because participants may not explicitly acknowledge or describe change in their accounts, and sense‐making may shift subtly as opposed to dramatically (Wanat et al., 2024). Participants may also use different language to describe similar experiences. A further recognized challenge in longitudinal qualitative research is that what appears to be change across waves may reflect shifts in how participants narrate, interpret, or position their experiences, rather than substantive change in experience itself (Neale, 2021; Wanat et al., 2024). Due to the aforementioned challenges, it is important that researchers engage with intentional analytic design, strong reflexive practice and practical data‐management strategies to ensure that their analytical interpretations do not become flattened and retain credible representations of idiographic trajectories of sense‐making.

Analytical frameworks are useful to researchers as they can help guide the researcher systematically through large qualitative datasets (Braun & Clarke, 2006, 2021). The addition of multiple temporal points of data collection adds a further level of complexity when seeking to identify conceptual patterns across a data corpus. As such, this paper proposes flexible step‐by‐step guidance that researchers can follow when conducting a LIPA. The steps incorporate analytical procedures that are both specific to LIPA (e.g., exploring convergence and divergence across longitudinal experiential trajectories) and those (e.g., familiarization) that universally transcend traditional approaches of conducting IPA and other qualitative analytical approaches (e.g., reflexive thematic analysis, discourse analysis). A visual overview of the suggested stages of analysis can be found in Figure 2. To help illustrate these steps, I have inserted examples from the data collected in the aforementioned study exploring long‐term recovery from substance use.

**FIGURE 2:**
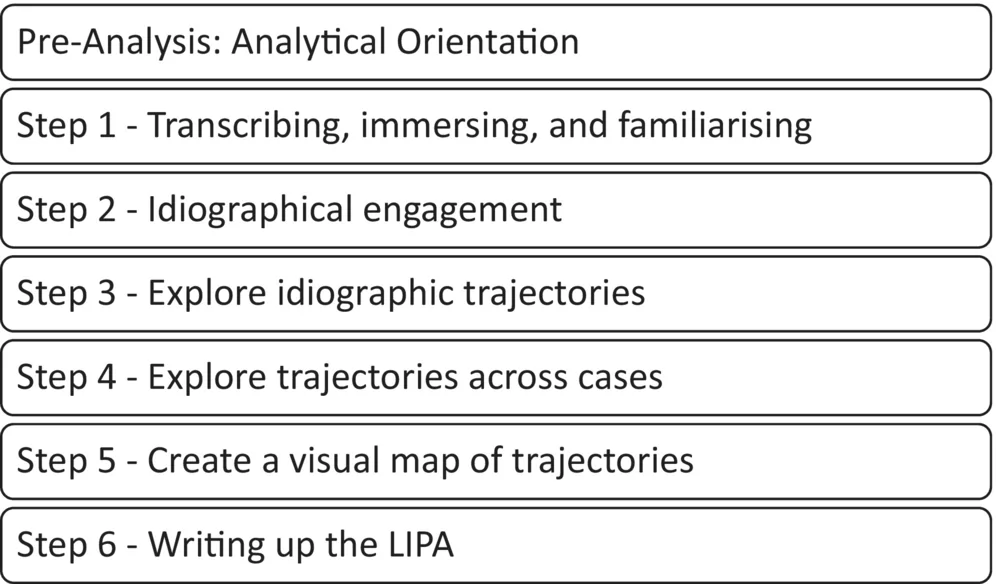
Analytical framework for LIPA.

### Pre‐analysis: Analytical orientation

Before engaging in any active coding, writing of experiential statements, or reflexive memos, researchers must first reflexively clarify their analytical intent. This will enable researchers to transparently consider what experiential change over time means for their specific study (longitudinal intent). During this step, researchers should also consider their analytical focus, determining whether their research is interested in individual trajectories, group trajectories, or a combination of both. Reflexive questions that may be considered during this pre‐analytical phase include: (i) ‘am I interested in change, stability, disruption, or *re‐meaning*?’, (ii) ‘what do I consider time to be? (e.g., event‐based, developmental, cyclical, or open‐ended)’ and (iii) ‘does my research question require me to focus more on group‐level experiences or individual trajectories?’ To add further clarity to the complex reflexive action of *re‐meaning*, I use this term to refer to the evolving interpretation of lived experience, whereby participants revisit and reconfigure the meaning of earlier experiences over time.

The purpose of this reflexive activity is to challenge and record any pre‐existing assumptions held by the researcher and to focus the researcher on the longitudinal scope specific to their study's aims and objectives.

### Step 1 – Transcription, immersing and familiarization

When adopting longitudinal approaches to qualitative analysis, researchers will have a large amount of rich idiographic data that has been collected at multiple time points. As such, before the researcher attempts to make sense of the data, they must familiarize themselves with the context, content and tone of the data. When data are collected using more traditional approaches such as semi‐structured interviews and focus groups, the act of immersion often begins with the initial transcription of audio‐recorded data. With the increasing use of videoconferencing software (e.g., Microsoft Teams or Zoom) to support data collection practices, many researchers are now using the built‐in transcription features provided by these platforms. In these instances, researchers should ensure they perform a quality check of the recording to ensure that conversations have been transcribed accurately, amending where necessary. The process of transcribing data allows researchers to listen to the conversations and interactions that took place and be reminded of the topics discussed. Furthermore, researchers can also annotate and record non‐verbal features of their interactions with participants, such as pauses, emphasis and emotive responses that can often be forgotten during the later stages of analysis.

Where data collection provides a written as opposed to spoken form of data, such as responses to open‐ended questionnaires, reflective journals and blogs, the researcher must also find ways to refamiliarize and immerse themselves with the data. This process may include multiple readings of the data and reflecting on the contextual factors that may have influenced a participant's response. Where participatory data collection methods such as walking interviews are used, researchers may wish to retrace the route taken during the interview while listening to the recording of the interview to be reminded of any spatial prompts within the conversation.

### Step 2 – Idiographically engage with the data

To retain the idiographic values of LIPA, it is suggested that researchers should analyse their data on a case‐by‐case basis, ensuring that they analyse each of the time points in chronological order for one participant before proceeding to the next. To familiarize themselves with each participant's data, researchers should read the data provided by the participant in the chronological order that it was collected. Once completed, researchers can begin to construct small, meaningful codes as annotations to help describe the data working sequentially across timepoints for each participant. Practically, during this stage researchers should clearly record the data collection interval each code is attributed to either in a table or case file, as this will help organize later stages of the analytical process. The concept of a code may often seem ambiguous to novice qualitative researchers. As such, it can be helpful to liken a code to an individual piece of a jigsaw. Alone, codes hold little meaning, but when pieced together, they construct patterns of conceptual meaning and sense‐making that provide rich and idiographic responses to experiential research questions.

Smith (2019) suggests that the search for meaning is a complex process for both participants and researchers, requiring both cognitive and emotional energy. Meaning can be situated at several levels, including literal meaning, pragmatic meaning, experiential meaning and existential meaning (Smith, 2019). As such, when attempting to code how the participant makes sense of their lived experience, the researcher may want to situate sense‐making within the broad analytical lifeworld concepts of temporality, spatiality, emotions and intersubjectivity (Larkin et al., 2019). Researchers may also identify a combination of lenses within their participants' experiences. For example, a participant may recount an experience where they have interacted with another person, such as a nurse or consultant (intersubjective lens), during their illness and found them to be frustrating (emotional lens).

### Step 3 – Explore idiographic trajectories

Once researchers have chronologically coded all data provided by a participant across all time points, they should work iteratively across these time points to identify whether the participant's sense‐making of the phenomenon under investigation remains stable or is dynamic and to identify any tensions or ambivalence within their accounts. To assist in this task, researchers should consider if their sense of emotion, spatiality and intersubjective sense‐making is dynamic or continuous. By the end of this stage, the researcher should have an in‐depth, longitudinal and idiographic case study highlighting experiential trajectories for each person within their sample.

An example from (Cornwall, 2025; Keenan et al., 2026) demonstrates how individuals dynamically made sense of their long‐term recovery from substance misuse. We identified that participants dynamically navigated their sense of shame over 1 year between interviews. When we first interviewed Simon, he expressed his guilt and shame for how he treated his mother, and especially for not being present in his daughter's life while she was growing up. He describes some of his feelings when the opportunity to reconnect came in the following account:

I remember in rehab just getting a letter saying from social services because she was only 14 that she wanted to see me, and I thought that it would be a long process, but they set it up for me to meet up with her and with her keyworker. I went there full of shame, how I abandoned her and because of what I had done during my recovery and rehab. I wanted to take responsibility and to admit my failures as a dad. When we got to Vicky (daughter), I just wanted to run because that's all I ever did was just run, you know. I was a coward because it was normal for me to run away from the important things in life. I kind of chose my path from [the age of] 16, to do what I want, and so I met her. Before I got to the meeting, I thought she is going to throw all of the things from the past at me. She didn't, she hugged me, and that was the start of our relationship. [Simon, 10th year of recovery – Time point 1]

When we met with Simon a year later for the second interview, he presented with a greater sense of maturity. Simon discussed how he had begun to translate his felt sense of shame into a will to make amends with people he had hurt during his time abusing substances:

For me, it's not all about just relinquishing some shame. It is actually about making amends to the people I've harmed or wrongdoings that I've done through my life. And then, not just, not even a recovery, I'm not perfect, I made mistakes, but it's having that self‐reflection and using the steps to do a self‐examination and take responsibility for my part when I've had shortcomings, even on a day‐to‐day basis. [Simon, 11th year of recovery – Time point 2]

In the second interview, Simon discussed how he had started to think more interpersonally about his recovery. The prior intrinsic focus on the need to relinquish shame had been translated to a more extrinsic and volitional focus on making amends to the people in his life who were affected by his prior substance misuse. Temporally, it was interpreted that Simon had developed a sense of maturity through this transference from an intrinsic to an extrinsic outlook during his recovery.

Using the example of Simon, his trajectory narrative encompasses a sense of dynamism through his reflexive action of *re‐meaning* his embodied sense of guilt and shame to a sense of reflection and volitional attempts to make amends for his past substance use through a process of maturation. Furthermore, Simon had begun to shift the orientation of his focus from one that was introspective in nature to one that considered how his past substance misuse had impacted the people that he loved and cared for. The trajectory summaries for each participant are useful as they act as an *aide‐mémoire* to analysts when navigating large datasets in the search of meaning. The summaries are the product of in‐depth analytical engagement with the data provided by the participant across all time points of data collection. As such, they help to condense large amounts of analytical coding into a narrative that can be more easily compared with those of other participants.

### Step 4 – Compare trajectories across cases

Once the researcher has identified trajectories for each of their participants, they can begin to explore the level of convergence and divergence with other participants across the data corpus to identify experiential trajectories that span across the entirety of the dataset. In the example study focusing on long‐term recovery from substance use, one posited trajectory spanning the entire data corpus was labelled *From Introspection to Authenticity*. Across this group‐level trajectory individuals within the sample were interpreted as being positioned at three distinct points, (i) *introspection*, (ii) *maturation* and (iii) *authenticity*. The position of introspection centred on a person's ability to discuss and find meaning in how they came to become dependent on substances (e.g., childhood trauma) and through what they had learned about themselves during treatment to help them sustain their recovery (e.g., development of new coping strategies). The position of maturation was centred on the same reflexive foundations as the position of introspection. However, individuals positioned at this stage of the trajectory also possessed the reflexive ability to see how their historic substance use had impacted others in the period of active substance‐use, treatment and during recovery. Thus, interpretations could reflect both inwardly and outwardly on the relative impacts of their substance use and consequently found themselves able to repair existing relationships and build new ones centred on trust. Finally, the position of authenticity represented individuals who continued to demonstrate reflexive awareness but had reached a point of acceptance of their ‘past‐selves’ and presented a new sense of identity built strongly upon the foundations of the learnings gained through navigating their recovery.

To help illustrate this level of cross‐case analysis, Simon the participant introduced in the preceding analytical step was positioned as being at the point of *maturation*. To help illustrate the objectives of this analytical step, I will introduce two further participants who were positioned at the other identified points across the experiential trajectory, Sophie (*introspection*) and Peter (*Authenticity*).

At the start of the project, Sophie was 14 years in recovery from her substance misuse. During the initial interview, Sophie reflected inwardly on her upbringing as a child in relation to what underpinned her problematic use of alcohol, and how she drank to enhance her perceived sense of confidence:

You know, they were really strong unmet needs in me like love and affection that weren't met when I was young, you know, without going into my mom's history as well but they just weren't met you know. So, yeah, it was hard, but I very much saw there was a sort of emotional emptiness, if you like, a lack of love, and deep feelings of shame, not being good enough, not being worthy, etc. It really fuelled my drinking, it gave me, you know, confidence, etc., around to the opposite sex, you know, to realize that was a big part of me drinking. You know, and it was a basis to my depression. [Sophie 14 years in recovery – time point 1]

A year later during our second interview, Sophie reflected more on how her experiences of long‐term recovery had shaped her understanding of herself. She discusses the components of her recovery that shape her sense of intrinsic identity, adaptability and resilience:

I think in terms of who I am and what I stand for, so that's my physical self, that's my emotional sense of who I am, my identity. It's my wholeness. It's almost been cemented more since the last time we spoke. But it's not tangible and that's the thing; it's got less and less tangible over the years. It's very much more an internal process, but it's very much something that I see is gelling together more and more and more especially over the last few months and probably the last year. Because, you know, studying through a pandemic and adapting to that, recognizing and building my resilience, my awareness, my sense of who I am as an individual, being unapologetically me as well and not kind of feeling that I have to censor myself in any way because I feel more aligned with who I am than I ever have done. [Sophie 15 years in recovery – Time point 2]

When comparing Sophie and Simon's individual trajectories it is clear that both are shaped by their engagement in an ongoing reflexive discussion that helps to sustain their recovery. However, there is also a level of conceptual and linguistic divergence. Simon had begun to translate his internal reflections to one that acknowledges the relative impact that his substance use had on others (notably his daughter) resulting in a desire to make amends. On the other hand, Sophie's internal reflections still had an inward utility, with a need to solidify an understanding and acceptance of herself, as demonstrated by her linguistic use of terms such as *unapologetically me* and not feeling a need to censor her developing sense of self.

Peter, at the time of the project, was 29 years in recovery from substance misuse. Similarly to Simon, Peter also demonstrated a sense of maturity through his reflections on his past substance misuse and recovery and how this impacted others. However, in contrast to both Simon and Sophie, Peter had been able to establish a greater level of authenticity in accepting his past and how this shaped his present and future identity:

I think to be reflective is a very important thing. But recently, I've come to realise that the benefit of reflecting on the past is that it allows you to move forward. It's not reflecting on the past and woe is me, glass of wine, Billie Holiday on the stereo. The past was great, but it will never be the same again. If you're reflecting on the past and taking the good things or the mistakes, looking at things and learning from them, it's a learning process, but it is ultimately about going forwards. It's not about wallowing in the past. [Peter, 29 years in recovery – time point 2]

In the interview with Peter, he appears to have a greater sense of resolution in terms of his shame and has actively reflected on the lessons he has learnt from his past substance misuse and recovery. Unlike Simon and Sophie, Peter's resolution has enabled him to situate himself in the present looking prospectively to the future. We interpreted Peter's reflections as solidifying an authentic sense of self, in a way that acknowledges the mistakes he had made and how they have allowed him to shape an authentic representation of himself moving forwards.

### Step 5 – Creation of a conceptual map visualizing experiential trajectories across the data corpus

Once the researcher has explored the convergence and divergence of experiential sense‐making longitudinally across the data corpus and identified the experiential trajectories, it is suggested that a conceptual map is created to visually represent them. This conceptual map may be used to cluster participants who are at similar temporal points in their experiential trajectories or share more convergence in their experiential sense‐making. For example, in our work (Cornwall, 2025; Keenan et al., 2026), the sample included participants who varied between 4 and 29 years in recovery following their problematic substance use. Although all participants shared experiential homogeneity, their experiential trajectories differed in relation to how they mapped onto the identified trajectories (see Figure 3).

**FIGURE 3:**
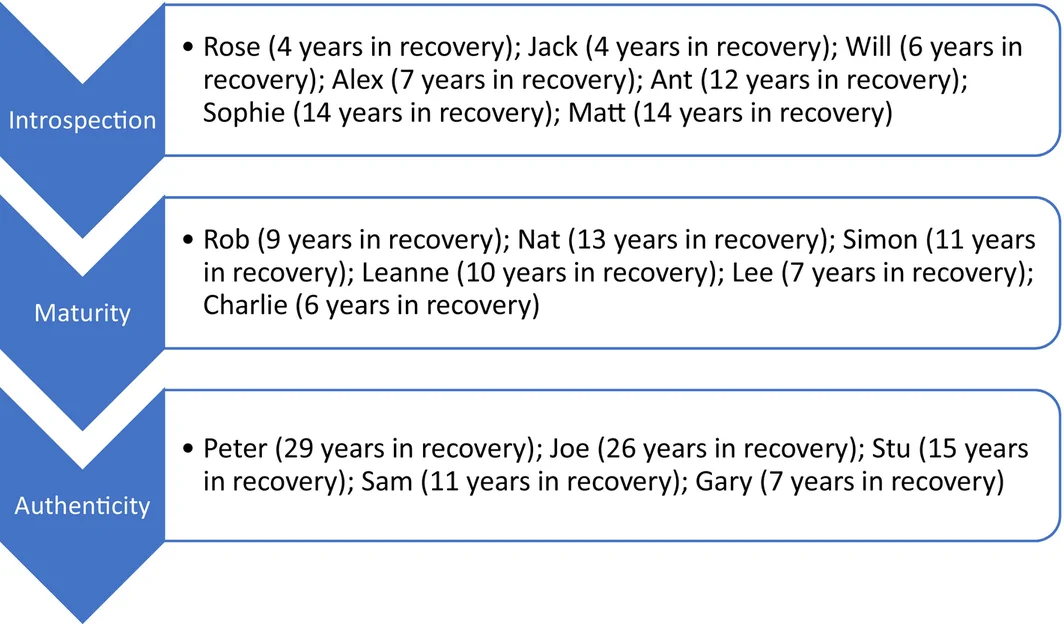
Mapping participants to identified experiential trajectories.

Through the study, a trajectory entitled *From introspection* to *authenticity* was interpreted. As such, it was possible to identify that long‐term recovery from substance misuse is a transformative experience that individuals dynamically navigate in terms of sense‐making. Along this trajectory, individuals were positioned at a point of introspection, where they are prioritizing maintaining sobriety and intrinsically understanding their sense of self, and a point of maturation, where they continue to engage in internal reflexive discussions but begin to externalize these to engage more relationally with others and make amends for their past misgivings. Finally, individuals can be positioned at a point in their recovery where they have self‐acceptance and are comfortable in expressing their authenticity. Individuals at this position accepted their past mistakes and acknowledge them as shaping their sense of identity both in the present and the future.

At this step of the analysis, it is also important to share a cautionary reminder about the scope of what can be achieved and claims that may be made when a LIPA has been utilized. Although LIPA may allow us to identify experiential trajectories and interpret the various positions that people may be placed along them, it is beyond the methodological scope of a LIPA to claim the speed and extent to which people will transition along an experiential trajectory.

### Step 6 – Writing up the LIPA

How best to present the findings of a LIPA will be determined by the analytical orientation outlined in the earlier phases. Farr and Nizza (2019) suggest that there are two ways of presenting the findings: (i) themes tied to time points or (ii) themes spanning time. Themes tied to time points organize findings around specific stages of the longitudinal process (e.g., Time 1, Time 2 and Time 3), highlighting how participants' experiences are understood at each point in time. In contrast, themes spanning time are organized around experiential patterns that extend across multiple time points, foregrounding continuity, change and the evolving meaning of participants' experiences over the course of the study. Using either approach, researchers should seek to present their findings in a conceptual manner that captures the idiographic and dynamic nature of the analysis conducted. To achieve this, researchers should present idiographic case summaries for each of their participants that identify their individual trajectories of sense‐making (step 3) before presenting conceptual patterns that have been identified at a group level.

Once completed, researchers can begin to report the conceptual trajectories that have been identified at the group level. Presenting longitudinal experiential themes guides the reader through the dynamic and static conceptual patterns identified across the data corpus. When considering titles for themes, researchers should not be limited to describing interpretations for individual time points. Rather, they should ensure that the fluidity or static nature of how individuals make sense of their lived experiences over the entire duration of an investigated social phenomenon is captured. Theme labels should be representative of longitudinal insights that respond relevantly to the research question(s), aims and objectives of a specific study.

The write‐up of each theme should also include extracts that evidence how sense‐making has changed temporally throughout the investigation. The researcher should weave case narratives using extracts from participants that clearly indicate interpretations of change or stability. Selecting the most appropriate extract from a rich and large data corpus is often a challenge for most researchers. To help determine which extracts may best illustrate the trajectories identified through the analysis, it is helpful to remind researchers that extracts serve a purpose as opposed to simply illustrating a theme. Therefore, researchers should consider using extracts that: (i) demonstrate continuity of meaning, (ii) demonstrate transformation of meaning or (iii) reveal tensions, contradictions, or ambivalence across time points. Where possible, researchers should prioritize sequenced extracts from the same participant (e.g., Simon's transformation from an intrinsic focus to an extrinsic one) in an attempt to demonstrate not only *what* has changed but, moreover, *how* the participants have derived meaning from the change. In terms of participant coverage, researchers do not need to include the voice of all participants in every theme. Rather, they should engage at a deeper level with fewer participants who act as exemplars of the conceptual cross‐case trajectories that have been used to construct each theme.

## CONCLUSION

This paper has provided a methodological discussion of practical and methodological considerations for researchers who are utilizing LIPA. It is also the first to provide a flexible framework that researchers can adapt and use to guide their analysis, depending on the longitudinal intent and analytic orientation of their specific research projects. The methodological considerations discussed in this paper apply to multiple research contexts, including health, education and broader social sciences.

Longitudinal phenomenological research provides a unique opportunity for researchers to explore the dynamic way individuals make sense of their lived experiences over time. It embraces the ideology that lived experiences do not occur in a vacuum and, as such, cannot be isolated from the prolonged contextual interactions that individuals are exposed to in the societies and communities in which they are embedded. Consequently, lived experiences must be interpreted in the context of an ever‐changing and dynamic world. As such, this paper argues that interactions and contextual factors shape and change the way we make sense of the world around us.

The adoption of a longitudinal approach to phenomenological research has the potential to enhance trustworthiness and authenticity. There are several different frameworks that qualitative researchers use to establish rigour within their research (Guba, 1981; Johnson & Waterfield, 2004), which outline the importance of demonstrating credibility, dependability, transferability, confirmability and criticality when conducting research. It is argued that LIPA enables researchers to demonstrate credibility through their prolonged immersion in the cultural or societal phenomenon under investigation (Shenton, 2004). The use of multiple time points in data collection also provides an opportunity to enhance credibility for qualitative researchers through member‐checking, triangulation and asking iterative questions (Shenton, 2004). It is hoped that the analytical framework suggested in this paper can help researchers demonstrate dependability in their research by suggesting different tasks that must be achieved during the analytical process.

However, as with all approaches to qualitative analysis, there are limitations to the use of longitudinal approaches in phenomenological research. Practically, LIPA is more time‐consuming and requires greater levels of cognitive analytic load to complete in comparison to other contemporary methods of qualitative research. To overcome this, consideration needs to be made early in the planning process whether longitudinal phenomenological research is feasible within a given time and whether there are appropriate analytical resources to support the project. Furthermore, there are methodological limitations, such as the risk of participant drop‐out during intervals between data collection and the potential for additional burden on participants due to having to engage with data collection at multiple intervals. To overcome this, before recruitment, participants should be fully informed as to the longitudinal aims and objectives of the research project. Expectations of participants should also be communicated to ensure that participants can provide their fully informed consent to participate at each interval of data collection.

In conclusion, this paper provides an important discussion of key methodological and analytical issues that must be considered when researchers plan to conduct longitudinal phenomenological research. It is important to note that the analytical framework outlined in this paper should not be viewed as an unadaptable and linear analytical pathway. Researchers using LIPA are encouraged to adapt this approach to one that best aligns with the analytical orientations and the aims and objectives of their study. This paper also does not seek to critique phenomenological research conducted at a single time point. Moreover, the considerations discussed in this paper are suggested as a companion or guide for researchers who are exploring how participants navigate their lived experiences over a prolonged period.

## AUTHOR CONTRIBUTIONS

**Joseph Keenan:** Conceptualization; investigation; writing – original draft; methodology; validation; visualization; writing – review and editing; software; formal analysis; project administration; data curation; resources.

## CONFLICT OF INTEREST STATEMENT

The author declares no conflict of interest.

## Data Availability

Data sharing not applicable to this article as no datasets were generated or analysed during the current study.
